# Clinical Phenotype, Molecular Architecture, and Survival Follow-Up in NRAS- and KRAS-Mutated Juvenile Myelomonocytic Leukemia

**DOI:** 10.3390/cancers18142236

**Published:** 2026-07-12

**Authors:** Bang Zhang, Chenmeng Liu, Xiaolan Li, Yang Wan, Xiaojuan Chen, Ye Guo, Li Zhang, Yao Zou, Fang Liu, Yumei Chen, Tianyuan Hu, Yingchi Zhang, Xiaofan Zhu, Wenyu Yang

**Affiliations:** 1 State Key Laboratory of Experimental Hematology, National Clinical Research Center for Blood Diseases, Haihe Laboratory of Cell Ecosystem, Institute of Hematology & Blood Diseases Hospital, Chinese Academy of Medical Sciences & Peking Union Medical College, Tianjin 300020, China; zhangbang@student.pumc.edu.cn (B.Z.);; 2 Tianjin Institute of Health Science, Tianjin 301600, China

**Keywords:** juvenile myelomonocytic leukemia, NRAS, KRAS

## Abstract

Background: Juvenile myelomonocytic leukemia (JMML) is a rare RAS/MAPK-driven pediatric myelodysplastic/myeloproliferative neoplasm. The phenotype and survival relevance of NRAS/KRAS alterations remain difficult to interpret because of co-mutations, evolving sequencing, incomplete germline confirmation, and post-diagnostic HSCT. Methods: We retrospectively reviewed 34 children with JMML and classifiable NRAS and/or KRAS alterations diagnosed between November 2010 and September 2022. Patients were classified as NRAS-only, KRAS-only, or NRAS/KRAS co-mutated. Direct comparisons used single-subtype cases. OS was analyzed in evaluable patients, with HSCT modeled as a time-dependent covariate. Results: The cohort included 20 NRAS-only, 12 KRAS-only, and 2 NRAS/KRAS co-mutated cases. KRAS-only cases had higher monocyte percentage (27.2% vs. 16.7%; *p* = 0.023) and lymphocyte percentage (48.1% vs. 39.0%; *p* = 0.018), whereas absolute monocyte count was comparable (4.0 vs. 4.0 × 10^9^/L; *p* = 0.930). PTPN11 was the most frequent non-RAS co-mutation (7/34) and occurred in both NRAS/KRAS co-mutated cases. Methylation status was available in 11 patients and analyzed descriptively. NRAS/KRAS subtype did not significantly stratify OS (HR for KRAS-only vs. NRAS-only, 0.55; 95% CI, 0.18–1.62; *p* = 0.276). HbF did not significantly stratify OS, while diagnostic total hemoglobin showed only an exploratory univariable association. Exact HSCT dates were retrieved for all 11 transplanted patients; time-dependent Cox analysis showed a significant association between HSCT and better OS (HR, 0.11; 95% CI, 0.02–0.56; *p* = 0.007). Conclusions: NRAS/KRAS status defined a limited diagnosis-time phenotype but did not independently stratify survival. JMML survival analyses should integrate co-mutations, germline and testing-era limitations, HbF/hemoglobin risk context, and time-dependent HSCT handling.

## Simple Summary

Juvenile myelomonocytic leukemia (JMML) is a rare childhood myeloid neoplasm driven by abnormal RAS/MAPK signaling. We reviewed 34 children with NRAS and/or KRAS alterations to clarify diagnostic clinical features, molecular context, and survival follow-up. NRAS-only and KRAS-only cases were broadly similar at diagnosis, NRAS-only and KRAS-only cases were broadly similar at diagnosis, although KRAS-only cases had higher relative monocyte and lymphocyte percentages without higher absolute monocytosis. Patients undergoing HSCT showed better observed survival than those managed without HSCT, though this retrospective association requires cautious interpretation. PTPN11 co-mutation was documented in a subset of patients, including both NRAS/KRAS co-mutated cases. Molecular testing evolved from hotspot-focused sequencing before 2017 to broader full-gene/next-generation sequencing thereafter. Patients undergoing HSCT showed better observe requires cautious interpretation. Overall, NRAS/KRAS status should be interpreted together with co-mutations, testing era, germline predisposition, and treatment context.

## 1. Introduction

Juvenile myelomonocytic leukemia (JMML) is a rare clonal myelodysplastic/myeloproliferative neoplasm of early childhood. Contemporary clinician-oriented reviews describe JMML as a biologically heterogeneous disease in which diagnosis, risk assessment, and treatment decisions require integration of clinical phenotype, hematologic findings, and molecular context [1,2]. The characteristic clinical picture includes persistent peripheral blood monocytosis, splenomegaly, cytopenia or leukocytosis, and abnormal myeloid progenitor responsiveness to granulocyte–macrophage colony-stimulating factor; these features have been emphasized in comprehensive reviews and classic clinical descriptions [3,4]. The current WHO and International Consensus Classification frameworks further reinforce that molecular data should be interpreted alongside clinicopathologic findings rather than in isolation [5,6].

The biologic basis of JMML is closely linked to dysregulated RAS/MAPK signaling [7,8]. DNA methylation profiling provides an additional prognostic layer: genome-wide methylation studies have shown outcome relevance, RAS-pathway mutation patterns have been linked to epigenetic subclasses, and international consensus definitions of methylation subgroups have been proposed [9,10,11]. These observations support the joint assessment of NRAS/KRAS alterations, co-mutations, methylation results, cytogenetics, treatment records, and survival follow-up, but they do not remove the need to report exactly how many patients had each measurement.

Within this framework, NRAS- and KRAS-mutated JMML deserve focused analysis because they form clinically recognizable subsets of the broader RAS-driven disease spectrum. Single-center retrospective series, however, often contain unevenly available specialized testing and follow-up. The value of such a cohort depends less on pretending that every variable is complete and more on reporting each result with the correct denominator.

For this reason, the present study was designed as a cohort analysis with explicit denominators. Each comparison was restricted to patients with the relevant information; unrecorded measurements were kept distinct from negative or normal findings, and exploratory analyses were separated from conclusions that would require uniform testing or external validation.

We therefore asked four practical questions: whether NRAS-only and KRAS-only JMML differ in routinely available diagnostic variables; whether co-mutations, methylation, and cytogenetic annotations add clinically interpretable context beyond the NRAS/KRAS subgroup alone; how treatment categories, particularly HSCT, should be incorporated into survival follow-up without overstating causal inference in a retrospective cohort; and how testing-era differences, including hotspot-focused sequencing before 2017 and broader full-gene/next-generation sequencing from 2017 onward, should be considered when interpreting unrecorded co-mutations. The goal was to extract the most reliable signals supported by the available data while avoiding overfitting prognostic modeling in a small, rare-disease cohort.

## 2. Materials and Methods

### 2.1. Study Design, Patient Cohort, and Diagnostic Criteria

This retrospective single-center cohort study was conducted at the Institute of Hematology and Blood Diseases Hospital, Chinese Academy of Medical Sciences & Peking Union Medical College, Tianjin, China. Children with an institutional diagnosis of JMML and classifiable NRAS and/or KRAS alterations diagnosed between 25 November 2010 and 8 September 2022 were included. Clinical follow-up information was reviewed through January 2023 when available. The retrospective review was performed under an institutional pediatric hematologic disease cohort protocol approved by the local ethics committee. De-identified institutional case data were reviewed for demographic characteristics, clinical presentation, hematologic parameters, coagulation indices, erythroid and colony-forming assays, cytogenetic findings, molecular and methylation annotations, immunophenotyping, cytokines, biochemical measurements, treatment exposure, HSCT documentation, and follow-up outcomes. The report was prepared with reference to STROBE principles for observational research [12]. Direct identifiers and exact follow-up dates were not included in analytic tables, figures, or supplementary summaries.

Diagnostic eligibility was reviewed against accepted JMML criteria rather than inferred from mutation status alone. The criteria considered included absolute peripheral-blood monocytosis (monocyte count ≥ 1 × 10^9^/L), blasts and promonocytes < 20% in peripheral blood and bone marrow, absence of BCR::ABL1-positive chronic myeloid leukemia, and supportive clinical, laboratory, cytogenetic, or molecular features such as splenomegaly, elevated fetal hemoglobin for age, monosomy 7, or another clonal cytogenetic abnormality, GM-CSF hypersensitivity or colony-forming growth when tested, and RAS-pathway lesions involving PTPN11, NF1, CBL, NRAS, or KRAS [1,2,5,6]. The disease framework was therefore JMML first; the present analysis then further restricted eligibility to cases with classifiable NRAS and/or KRAS alterations so that NRAS-only, KRAS-only, and NRAS/KRAS co-mutated subsets could be defined consistently.

**Figure 1:**
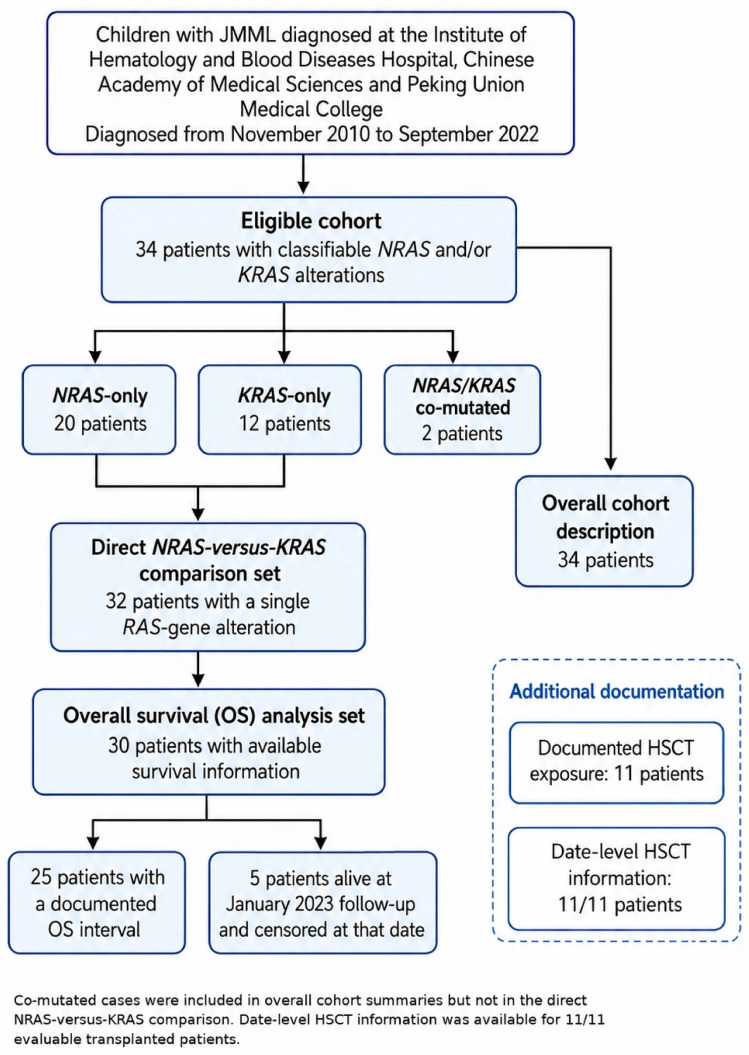
Analytic cohort and analysis sets. The flowchart summarizes cohort assembly, RAS-subgroup classification, survival-analysis eligibility, and HSCT documentation. Direct NRAS-versus-KRAS comparisons included 32 single-subtype patients, and OS analysis included 30 patients with analyzable survival information.

Cases were identified from institutional records on the basis of a clinical JMML diagnosis and a classifiable NRAS and/or KRAS alteration. The present analysis was not designed as an all-comers institutional JMML incidence series. Patients with other canonical JMML drivers but no classifiable NRAS/KRAS alteration, cases without retrievable molecular classification, and cases lacking sufficient documentation for a given analysis were outside the corresponding analytic denominator. Figure 1, therefore, reports the analytic cohort and analysis sets rather than a hospital-wide screened denominator.

### 2.2. Molecular Testing, Mutation Annotation, and Subgroup Definition

NRAS and KRAS mutation status was abstracted from diagnostic clinical molecular testing performed on available peripheral-blood or bone-marrow specimens as part of routine care. Molecular testing evolved during the study period. Before 2017, testing was primarily hotspot-focused sequencing and therefore had limited ability to exclude non-hotspot or broader cooperating alterations. From 2017 onward, broader full-gene/next-generation sequencing was adopted in routine diagnostic evaluation. Consequently, a missing or unrecorded co-mutation in earlier cases was not interpreted as a confirmed negative result. Source records were reviewed for the mutated gene, protein-level annotation, variant allele frequency when available, and non-RAS co-mutations when documented. Matched non-hematopoietic germline-control testing was not uniformly available across the long study period; therefore, mutations were reported as clinical molecular annotations unless germline or somatic origin was supported by the available records. Patients were grouped as NRAS-only, KRAS-only, or NRAS/KRAS co-mutated according to the detected RAS gene alterations. Comparisons between NRAS-only and KRAS-only groups included only patients with a single RAS-gene alteration. The two NRAS/KRAS co-mutated cases were included in the overall cohort description but were not included in direct NRAS-versus-KRAS comparisons, and no inference was made regarding clonal architecture or subclonal order.

DNA methylation status was curated from institutional reports when available. Because methylation testing was performed only in a subset of patients and platform, classifier, and cutoff details were not uniformly available in the curated dataset, methylation class was summarized descriptively with the tested denominator. It was not treated as a whole-cohort classifier, and methylation-related Cox estimates were interpreted only as exploratory tested-subset summaries.

### 2.3. Variables, Missing Data, and Analysis Sets

Clinical, laboratory, molecular, treatment, and follow-up data were extracted from institutional medical records. Baseline data included age at diagnosis, sex, presenting clinical features, blood counts, liver and spleen size, cytogenetic findings, NRAS/KRAS mutation status, non-RAS co-mutations, and DNA methylation results when available. Treatment and follow-up data included documented HSCT exposure, transplantation date when available, non-HSCT treatment information when retrievable, survival time, vital status, and cause of death when documented.

Missing data were not imputed. If a variable was not recorded in the medical record, it was treated as unavailable and was not considered negative, normal, or absent. This rule was especially important for molecular annotations because the pre-2017 hotspot-focused testing era could under-detect broader cooperating alterations compared with the broader full-gene/next-generation sequencing used from 2017 onward. For molecular and methylation annotations, a blank or unrecorded field indicated absence of a curated positive annotation or lack of retrievable testing documentation, not a confirmed negative result unless the source record explicitly supported that interpretation. Continuous variables were analyzed only in patients with available measurements. Categorical variables were calculated using the number of evaluable patients as the denominator.

Three analysis sets were defined before the analysis. The full cohort included 34 patients with clinically diagnosed JMML and classifiable NRAS and/or KRAS alterations. This cohort was used for overall clinical and molecular description. The direct NRAS-only versus KRAS-only comparison included 32 patients, after excluding the 2 NRAS/KRAS co-mutated cases. The overall survival (OS) analysis included 30 patients who had survival time and vital status, or a clearly defined censoring date.

### 2.4. Outcome Definitions and HSCT Handling

Overall survival (OS) was defined as the time from diagnosis to death from any cause or last confirmed follow-up. The follow-up cutoff date for this study was January 2023. Patients who were alive at the last confirmed follow-up were censored at that date. Because formal reverse Kaplan–Meier follow-up estimation could not be reconstructed from uniformly complete patient-level follow-up records, the manuscript reports the observed diagnosis-to-death-or-censoring interval and labels it explicitly as such rather than as a definitive cohort follow-up duration.

The OS analysis set included 30 patients. Among them, 25 patients had a directly documented OS interval in the medical records. Another 5 patients were confirmed to be alive at the January 2023 follow-up and were censored at that date. Five patients in the full cohort were recorded as lost to follow-up in the clinical records. The label “lost to follow-up” was not used as an outcome event. Patients recorded as lost to follow-up contributed to the OS analysis only if an analyzable survival interval or last confirmed follow-up date was available; otherwise, they were excluded from OS estimation. Patients without sufficient survival time or vital-status information were excluded from OS analysis but remained eligible for baseline and molecular description. The relationship between lost-to-follow-up status, censoring, and OS eligibility is summarized in Supplementary Table S1 and should be interpreted as a documentation audit rather than an endpoint.

Event-free survival (EFS), relapse/progression, treatment response, and cause-specific mortality were not used as primary endpoints because these events were not uniformly documented across the cohort. Treatment categories were curated as documented HSCT, wait-and-see, documented non-HSCT management, or treatment not documented. HSCT exposure was documented in 11 patients, and date-level transplantation information was available for all 11 documented HSCT-exposed patients. The median interval from diagnosis to HSCT was 183 days, corresponding to 6.0 months (IQR, 140–183.5 days; 4.6–6.0 months). Because HSCT is a post-diagnostic and non-random treatment exposure, HSCT-related survival comparisons were interpreted as observational treatment-context associations rather than causal estimates of transplant efficacy. Wait-and-see patients and patients without documented treatment category were not merged into the documented non-HSCT management group for the primary treatment-category survival comparison.

### 2.5. Statistical Analysis

Continuous variables were summarized as median and interquartile range (IQR), with the corresponding number of patients shown. Categorical variables were summarized as numbers and percentages among patients with available information. NRAS-only and KRAS-only groups were compared using the Mann–Whitney U test or Fisher’s exact test, as appropriate. OS was estimated by the Kaplan–Meier method, and group comparisons were performed using the log-rank test [13,14]. Unadjusted Kaplan–Meier curves were used to visualize observed survival by documented treatment category. Because HSCT was assigned during clinical care rather than randomly and occurred after diagnosis, the log-rank result from this unadjusted visualization was not used as the formal HSCT-related statistical result. The formal HSCT-related sensitivity analysis used Cox modeling with HSCT treated as a post-diagnostic time-dependent covariate, so transplanted patients contributed non-HSCT risk time before transplantation and HSCT-exposed risk time after transplantation. Exploratory Cox modeling was retained only as a transparency analysis and was not used to define new prognostic markers. *p* values were not adjusted for multiplicity and should be interpreted descriptively rather than as confirmatory evidence. All statistical analyses and figures were performed using Python (version 3.11).

## 3. Results

### 3.1. Cohort Assembly and Availability of Key Variables

The analytic cohort included 34 children with JMML and classifiable NRAS and/or KRAS alterations diagnosed at the study hospital between November 2010 and September 2022 (Figure 1). This was a mutation-defined analytic cohort rather than a complete institutional denominator of all JMML diagnoses during the same period. The cohort consisted of 20 NRAS-only patients, 12 KRAS-only patients, and 2 patients with both NRAS and KRAS alterations. Direct subtype comparison was restricted to the 32 single-subtype patients; the two co-mutated cases were retained for descriptive cohort summaries but not included in NRAS-only versus KRAS-only statistical testing.

Variable availability differed across measurements (Supplementary Table S1). Demographic, diagnosis, and core NRAS/KRAS variables supported whole-cohort description, whereas methylation, extended biologic testing, HSCT timing, and survival analysis used smaller evaluable denominators. Molecular data also reflected a testing-era transition: cases before 2017 were mainly evaluated by hotspot-focused sequencing, whereas cases from 2017 onward had broader full-gene/next-generation sequencing. This distinction is central to interpretation because unrecorded co-mutations, especially in earlier cases, cannot be assumed to be true negatives. The 34-patient cohort defines the clinical and molecular population, the 32-patient set defines the direct NRAS-only versus KRAS-only comparison, and the 30-patient set defines the OS analysis.

### 3.2. Baseline Clinical and Hematologic Phenotype

Median age at diagnosis was 14.5 (12.0–34.8) months, and 27/34 patients were male. Baseline hematologic abnormalities resembled the expected JMML presentation described in prior clinical literature, with anemia, leukocytosis, thrombocytopenia, and monocytosis frequently observed [1,3,4]. In this cohort, median diagnostic total hemoglobin concentration was 83.5 (77.0–97.0) g/L, WBC 20.9 (12.3–40.8) × 10^9^/L, platelet count 35.5 (21.5–74.0) × 10^9^/L, and monocyte percentage 19.0 (13.5–27.7) among patients with available values.

Baseline clinical and hematologic features were generally comparable between NRAS-only and KRAS-only cases. KRAS-only cases had nominally higher monocyte percentage than NRAS-only cases (27.2% vs. 16.7%, *p* = 0.023) and nominally higher lymphocyte percentage (48.1% vs. 39.0%, *p* = 0.018). However, the absolute monocyte count was not different between the two groups (median, 4.0 × 10^9^/L vs. 4.0 × 10^9^/L; *p* = 0.930). The observed monocyte-related difference should be interpreted as a difference in the relative proportion of circulating monocytes, not as evidence of a greater absolute monocyte count in KRAS-only disease. Because monocyte percentage is influenced by the total white blood cell count and the distribution of other leukocyte subsets, a higher percentage can occur even when the absolute monocyte count is comparable, as is shown in Table 1.

**Figure 2:**
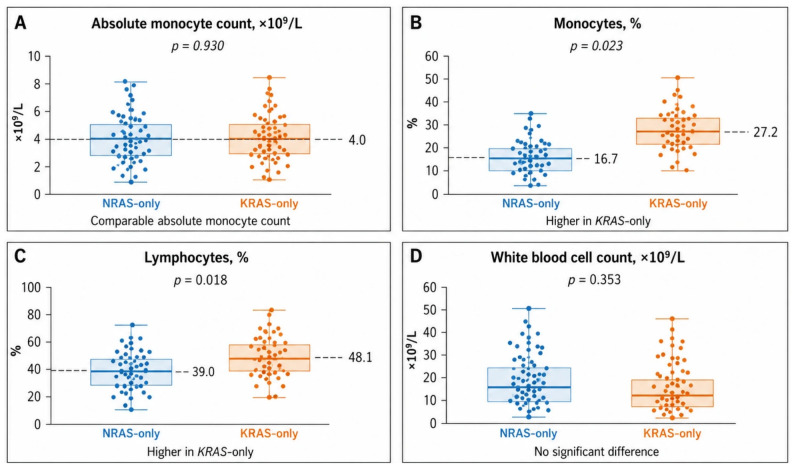
Baseline clinical and hematologic phenotype by RAS subgroup. (**A**) Absolute monocyte count in NRAS-only and KRAS-only cases. (**B**) Monocyte percentage in NRAS-only and KRAS-only cases. (**C**) Lymphocyte percentage in NRAS-only and KRAS-only cases. (**D**) White blood cell count in NRAS-only and KRAS-only cases. Box-and-dot plots show individual patient values and group distributions. *p* values were calculated using the Mann–Whitney *U* test and are exploratory.

These findings are notable but exploratory because the comparison involved small and variable-specific patient numbers and was not adjusted for multiple testing. Other routine baseline variables, including age, diagnostic total hemoglobin concentration, WBC, platelet count, spleen size, liver size, sex, lymphadenopathy, and rash, did not show nominally significant differences between the two single-subtype groups. The distribution of key baseline variables and the individual data points for each RAS subgroup are shown in Figure 2.

### 3.3. Molecular Architecture, Co-Mutations, Methylation, and Cytogenetics

The molecular annotations included both canonical hotspot and noncanonical or incompletely specified NRAS/KRAS protein changes, and the primary analysis therefore used gene-level NRAS/KRAS subgrouping rather than amino-acid-hotspot grouping. Protein-level annotations were interpreted conservatively so that amino-acid changes belonging to non-RAS genes were not assigned to NRAS or KRAS categories. Non-RAS co-mutations were identified in 11/34 patients, with PTPN11 being the most frequent accompanying alteration. This pattern is biologically plausible because somatic PTPN11 mutations are a recognized JMML lesion [15], and CBL alterations have also been repeatedly described in JMML and related predisposition settings [16,17]. Larger genomic studies have placed these lesions within a broader landscape that also includes secondary and cooperating alterations such as SETBP1 and JAK3 [18,19,20,21].

DNA methylation status was available in 11 patients, including 6 intermediate-methylation and 5 high-methylation cases. No low-methylation case was documented among the tested patients. These proportions describe only the tested subset and should not be interpreted as whole-cohort methylation prevalence. Cytogenetic information included normal karyotypes in many patients and monosomy 7 in selected cases. Because DNA methylation has prognostic relevance in JMML but was incompletely tested here, these results were summarized descriptively with the tested denominator rather than treated as a whole-cohort classifier [9,10,11].

**Figure 3:**
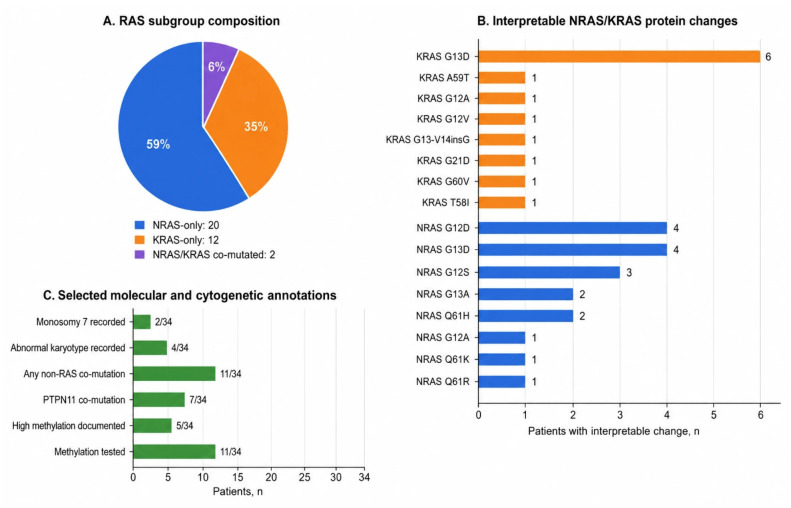
(**A**) RAS subgroup composition in the 34-patient cohort. Blue indicates NRAS-only cases, orange indicates KRAS-only cases, and purple indicates NRAS/KRAS co-mutated cases. (**B**) Interpretable NRAS/KRAS protein-level alterations summarized by patient count. Blue columns indicate NRAS protein changes, and orange columns indicate KRAS protein changes. (**C**) Selected molecular and cytogenetic annotations summarized by patient count. Green columns indicate the number of patients with each documented annotation, including methylation testing availability, high methylation status, PTPN11 co-mutation, any recorded non-RAS co-mutation, abnormal karyotype, and monosomy 7. The colors are used only to distinguish RAS subgroups or annotation categories and do not indicate statistical significance.

**Figure 4:**
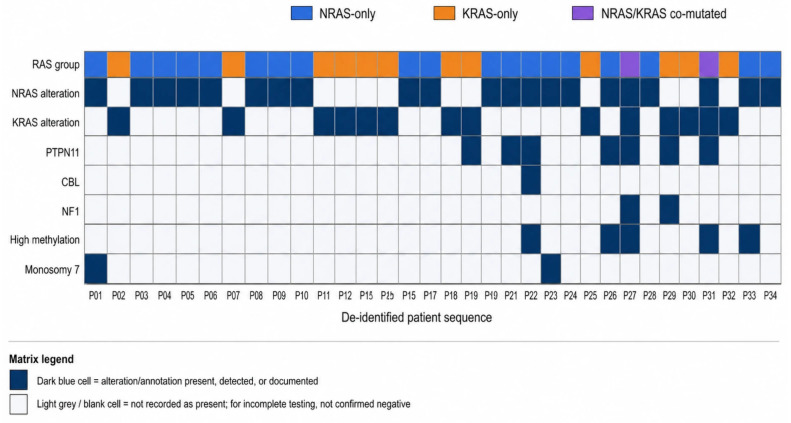
Patient-level core molecular annotation matrix. Each column represents one de-identified patient. The top colored bar indicates RAS subgroup membership: blue denotes NRAS-only, orange denotes KRAS-only, and purple denotes NRAS/KRAS co-mutated disease. Dark blue cells indicate that the corresponding alteration or annotation was detected or documented. Light gray or blank cells indicate that the corresponding positive annotation was not recorded in the curated dataset; for variables with incomplete testing, blank cells should not be interpreted as confirmed negative results. The matrix was simplified to focus on core NRAS/KRAS alterations, selected JMML-relevant co-mutations, methylation status, and monosomy 7; exploratory low-frequency rows and treatment/outcome rows were removed from the main matrix.

Detailed NRAS/KRAS protein-level annotations and selected molecular and cytogenetic annotations are summarized in Figure 3 and Supplementary Tables S2 and S3. Patient-level core molecular annotations are shown in Figure 4. Figure 3 now summarizes only RAS subgroup composition, interpretable NRAS/KRAS protein-level alterations, and selected major molecular/cytogenetic annotations; Figure 4 now focuses on core NRAS/KRAS alterations, selected JMML-relevant co-mutations, methylation status, and monosomy 7. Low-frequency exploratory co-mutations and treatment/outcome rows were retained only in the Supplementary Materials or text where appropriate, rather than emphasized in the main figure panels. Both NRAS/KRAS co-mutated patients had documented PTPN11 co-mutation; however, no earlier historical samples were available for these patients, so the temporal order of NRAS, KRAS, and PTPN11 acquisition could not be reconstructed. The cohort-level molecular, cytogenetic, methylation, treatment, and follow-up summary is provided in Table 2.

### 3.4. Extended Biologic and Laboratory Indicators

Several additional measurements were available in subsets of patients and help place the cohort in a biologic context. These variables did not create additional validated endpoints, but they strengthened patient-level annotation by capturing erythroid activity, colony-forming readouts, coagulation status, immunophenotyping, cytokines, immunoglobulins, complement, ferritin, EPO, bilirubin, LDH, and related laboratory features.

The value of these extended measurements is descriptive rather than inferential. They show that NRAS/KRAS-mutated JMML cannot be reduced to the mutation gene alone, but they were measured unevenly and therefore should not be promoted as independent prognostic markers without external validation. The extended biologic feature heatmap is provided in Supplementary Figure S1, and detailed extended biologic and laboratory variables are provided in Supplementary Table S5. These materials were used to display biologic breadth and missingness, not to imply complete measurement across the cohort.

### 3.5. Treatment Exposure, HSCT Documentation, and Follow-Up Audit

**Figure 5:**
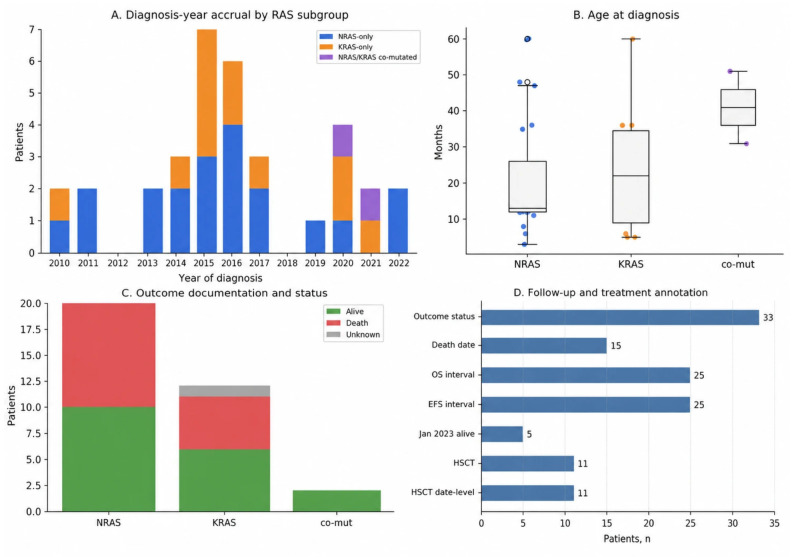
Patient-level diagnostic and follow-up documentation. (**A**) Stacked bars show diagnosis-year accrual by RAS subgroup; colors follow the RAS subgroup scheme used in Figure 3 and Figure 4. (**B**) Box-and-dot plots show age at diagnosis; colored dots represent individual patients, with dot colors corresponding to RAS subgroup. (**C**) Stacked bars summarize outcome documentation and vital status; green, red, and gray indicate alive, death, and unknown status, respectively. (**D**) Blue bars summarize availability of follow-up and treatment annotations, including date-level HSCT information. The color coding is descriptive and does not indicate statistical significance.

Treatment, HSCT, and follow-up information were reviewed in the 34-patient cohort. Documented treatment categories were HSCT in 11 patients, wait-and-see in 3 patients, documented non-HSCT management in 17 patients, and treatment not documented in 3 patients. Exact transplantation dates were retrieved for all 11 patients with documented HSCT; the median diagnosis-to-HSCT interval was 183 days, corresponding to 6.0 months (IQR, 140–183.5 days; 4.6–6.0 months). The wait-and-see group was kept separate from documented non-HSCT management, and patients without a documented treatment category were not merged into the documented non-HSCT group. Patient-level treatment documentation and HSCT timing are summarized in Figure 5. Because HSCT is a post-diagnostic, non-random intervention, Section 3.6 presents the treatment-category Kaplan–Meier curve only descriptively and uses time-dependent Cox modeling as the formal HSCT-related analysis. De-identified patient-level clinicomolecular and outcome categories are provided in Supplementary Table S4.

The follow-up cutoff date was January 2023. Fifteen deaths were documented during follow-up. Cause-of-death categories were not uniformly available and were therefore not analyzed. Five patients were recorded as lost to follow-up in the clinical records. Overall survival analysis was restricted to patients with available survival time and vital status or a defined censoring date. Thirty patients met these criteria and were included in the OS analysis. This analysis set included 25 patients with a documented OS interval and 5 patients who were alive at the January 2023 follow-up and were censored at that date. The median observed diagnosis-to-death-or-censoring interval in the OS analysis set was 11.5 months (IQR, 4.7–19.0; range, 0.0–102.0).

Detailed availability of clinical, laboratory, treatment, and follow-up variables is provided in Supplementary Table S1. The patient-level documentation overview in Figure 5 was used to show treatment-category counts and HSCT timing, not to imply uniform availability of all post-treatment endpoints.

### 3.6. Overall Survival and Exploratory Prognostic Analysis

Overall survival (OS) was analyzed in 30 patients with available survival time and vital status or a defined censoring date. Fifteen deaths were documented. The median observed diagnosis-to-death-or-censoring interval was 11.5 months, and the median OS was 13.0 months. Cause-specific mortality, relapse-related mortality, and transplant-related mortality were not analyzed because cause-of-death and post-treatment event documentation were not uniformly available.

**Figure 6:**
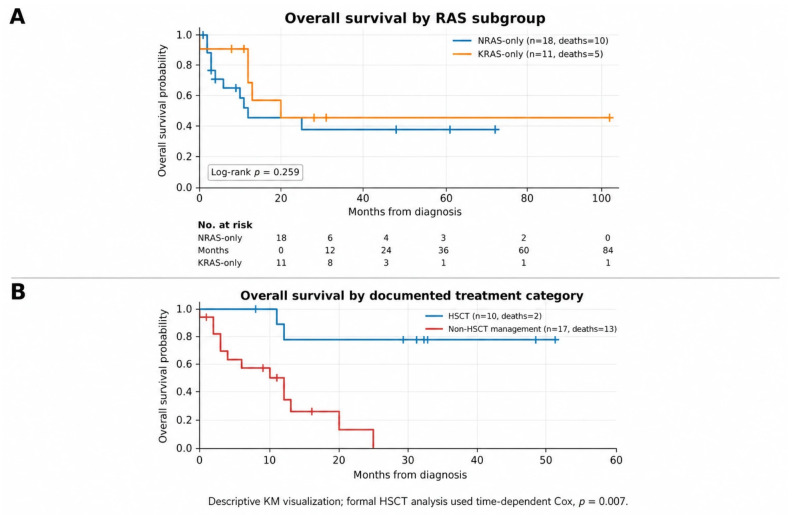
Overall survival by RAS subgroup and documented treatment category, with formal HSCT inference handled as a time-dependent exposure. (**A**) Kaplan–Meier curves compare OS between NRAS-only and KRAS-only patients in the OS analysis set. The NRAS-only group included 18 patients with 10 deaths, and the KRAS-only group included 11 patients with 5 deaths. Median OS was 11.0 months in the NRAS-only group and 20.0 months in the KRAS-only group; log-rank *p* = 0.259. Exploratory Cox analysis showed that KRAS-only status was not significantly associated with OS compared with NRAS-only status (HR, 0.55; 95% CI, 0.18–1.62; *p* = 0.276). (**B**) Kaplan–Meier curves show observed OS by documented treatment category and are retained as a descriptive visualization only. The formal HSCT-related statistical result was the time-dependent Cox sensitivity analysis: patients contributed non-HSCT risk time before transplantation and HSCT-exposed risk time after transplantation, and HSCT as a time-dependent covariate was significantly associated with better OS (HR, 0.11; 95% CI, 0.02–0.56; *p* = 0.007). Because HSCT is a post-diagnostic, non-random treatment exposure, the descriptive Kaplan–Meier separation should not be interpreted as randomized causal evidence of transplant efficacy.

NRAS-only and KRAS-only patients did not show a statistically significant OS difference (Figure 6A). The NRAS-only group included 18 patients with 10 deaths, whereas the KRAS-only group included 11 patients with 5 deaths. Median OS was 11.0 months in the NRAS-only group and 20.0 months in the KRAS-only group. The estimated 12-month OS rates were 40.1% and 68.2%, and the 24-month OS rates were 40.1% and 42.6%, respectively. In univariable Cox analysis, KRAS-only status was not significantly associated with OS compared with NRAS-only status (HR, 0.55; 95% CI, 0.18–1.62; *p* = 0.276). These findings indicate that the NRAS/KRAS subtype alone did not provide statistically significant OS stratification in this cohort.

HbF percentage was reviewed separately from diagnostic total hemoglobin concentration because elevated age-adjusted HbF is a recognized risk-related feature in JMML [1,2]. In this cohort, HbF data were incomplete and did not show a statistically significant univariable association with OS in the evaluable subset; therefore, HbF was retained as an established risk-context variable rather than as an independently validated predictor from this dataset.

Although diagnostic total hemoglobin showed an exploratory univariable Cox signal in this small cohort, it was not advanced as a validated prognostic marker. Total hemoglobin at diagnosis can be influenced by transfusion exposure, marrow failure, disease phase, inflammatory status, diagnostic timing, and subsequent treatment selection; therefore, this signal was treated as hypothesis-generating and was not used to override established JMML risk factors.

The documented HSCT/non-HSCT Kaplan–Meier curve was retained in Figure 6B to show the observed treatment-category survival separation, because removing the survival image would obscure a clinically important pattern. However, this curve was explicitly labeled as descriptive only and was not used as the formal HSCT-related statistical inference. Among patients with documented treatment category and analyzable survival, the HSCT group had 2/10 deaths and median OS was not reached, whereas the documented non-HSCT management group had 13/17 deaths and median OS was 10.7 months. Because HSCT occurred after diagnosis, the formal HSCT-related result was taken from a focused time-dependent Cox sensitivity analysis: in the documented HSCT versus documented non-HSCT treatment-comparison set, HSCT as a time-dependent covariate was significantly associated with better OS (HR, 0.11; 95% CI, 0.02–0.56; *p* = 0.007). This statistically significant association was interpreted cautiously because HSCT is a post-diagnostic, non-random treatment exposure and because treatment selection may reflect baseline risk, disease course, donor availability, clinical stability, and survivorship before transplantation. Wait-and-see patients and patients without a documented treatment category were described separately and were not merged into the documented non-HSCT group for this comparison. Numerical OS estimates for the RAS subgroups and documented treatment categories are provided in Supplementary Table S6, and HSCT timing for the time-dependent exposure definition is summarized in Supplementary Table S7.

**Figure 7:**
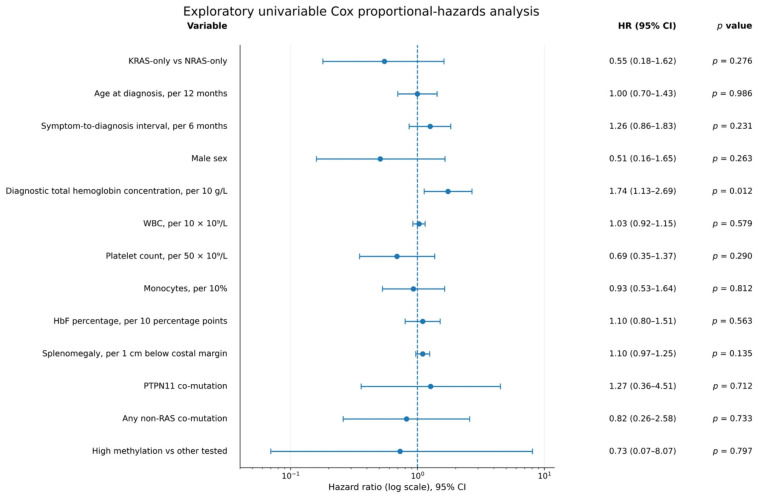
Exploratory univariable Cox proportional-hazards analysis. Hazard ratios are shown on a logarithmic scale with 95% CIs. Each blue dot represents the point estimate of the hazard ratio for the corresponding variable, and the horizontal blue line represents the 95% CI. The vertical dashed line marks HR = 1.0. Variables with insufficient data/events were not plotted. Diagnostic total hemoglobin concentration and HbF percentage were analyzed as separate variables. These exploratory estimates were retained for transparency and were not used to define new prognostic markers.

Exploratory univariable Cox estimates were retained only as a transparency analysis and are displayed in Figure 7. These estimates were not used to define new prognostic markers or to override established JMML risk factors. Detailed OS estimates by RAS subgroup and treatment category are provided in the revised supplementary tables. The corresponding exploratory Cox estimates are summarized in Table 3.

### 3.7. Integrated Result Summary

NRAS-only and KRAS-only patients showed broadly similar baseline clinical features. KRAS-only patients had nominally higher monocyte and lymphocyte percentages, whereas the absolute monocyte count and diagnostic total hemoglobin concentration were comparable between groups.

Molecular findings were consistent with RAS-driven JMML, and non-RAS co-mutations were documented in a subset of patients. Both NRAS/KRAS co-mutated patients had documented PTPN11 co-mutation, but earlier historical samples were unavailable and the order of mutation acquisition could not be reconstructed. The 2017 transition from hotspot-focused sequencing to broader full-gene/next-generation sequencing was considered when interpreting unrecorded co-mutations.

OS did not differ significantly between NRAS-only and KRAS-only patients, supporting the conclusion that the NRAS/KRAS subgroup alone is insufficient for outcome interpretation in this cohort. In contrast, the documented treatment category showed a significant observed survival difference: patients undergoing HSCT had better observed OS than patients managed without HSCT. This treatment-category difference was interpreted cautiously because HSCT is a post-diagnostic, non-random intervention.

## 4. Discussion

This single-center study analyzed 34 children with clinically diagnosed JMML carrying NRAS and/or KRAS alterations. The revised analysis addresses several issues central to JMML interpretation: the separation of NRAS-only, KRAS-only, and NRAS/KRAS co-mutated groups; the distinction between clinical molecular annotations and uniformly confirmed somatic mutations; the potential relevance of germline RASopathy/Noonan-spectrum predisposition; the 2017 transition from hotspot-focused sequencing to broader full-gene/next-generation sequencing; and the need to interpret HSCT-associated survival differences in treatment context rather than as randomized causal effects.

The main exploratory clinical observation was a diagnostic blood-cell differential pattern in KRAS-only disease. KRAS-only patients had higher monocyte and lymphocyte percentages than NRAS-only patients, whereas the absolute monocyte count was comparable between groups. This indicates a difference in leukocyte differential composition rather than a greater absolute monocyte burden. Other baseline clinical and hematologic features were largely similar between groups, suggesting that NRAS/KRAS subtype alone does not define a distinct baseline clinical phenotype. Because multiple baseline variables were tested in a small cohort, these differences should be considered exploratory and hypothesis-generating.

This interpretation is consistent with previous JMML studies showing that RAS-pathway alterations are associated with clinical heterogeneity and that genotype–phenotype relationships are not determined by a single RAS gene alone [22,23,24]. Reports of spontaneous hematologic improvement, germline NRAS-associated disease, and spontaneous remission in selected RAS- or NRAS-mutated cases further support cautious interpretation of RAS subtype as a standalone clinical classifier [25,26,27].

The molecular findings were consistent with JMML as a RAS/MAPK-driven disease [7,8]. Non-RAS co-mutations were detected in a subset of patients, with PTPN11 being the most frequent accompanying alteration. The overall documented PTPN11 co-mutation rate was 7/34 (20.6%), and both NRAS/KRAS co-mutated patients harbored PTPN11 co-mutation. This observation is clinically interesting but must be interpreted with caution because testing methods changed over time and because the two double-mutated cases are too few for inference. Earlier historical samples were not available for the NRAS/KRAS/PTPN11 co-mutated patients, so whether one RAS-pathway lesion preceded the others could not be determined. Larger genomic studies have shown that JMML can include cooperating or secondary alterations, supporting integrated interpretation rather than reliance on NRAS/KRAS status alone [18,19,20,21].

DNA methylation has established prognostic relevance in JMML [9,10,11]. In this cohort, methylation data were available in only 11 patients and were therefore summarized descriptively. Because platform, classifier, and cutoff information were not uniformly available in the curated dataset, methylation should not be interpreted as a whole-cohort validated risk classifier in this report. Extended biologic and laboratory variables, including HbF, colony-forming assays, immunophenotyping, cytokines, immunoglobulins, complement, ferritin, EPO, and LDH, were also available only in subsets of patients. These variables enriched patient-level annotation but could not support independent prognostic conclusions.

This conservative interpretation is supported by studies showing that JMML biology extends beyond the primary RAS lesion, including genetic and epigenetic origins detectable early in life, fetal-like biologic programs, and CBL-associated molecular and phenotypic diversity [28,29,30].

Survival analysis did not show significant OS separation between NRAS-only and KRAS-only patients. This does not prove that RAS subtype has no prognostic relevance; rather, in this small cohort, NRAS/KRAS status alone did not capture the outcome heterogeneity created by co-mutations, methylation class, germline predisposition, treatment selection, and HSCT timing. The revised manuscript, therefore, avoids presenting NRAS/KRAS subtype as a standalone prognostic classifier.

HbF and diagnostic total hemoglobin concentration should be interpreted separately. Elevated HbF is a recognized adverse risk-related feature in JMML [1,2]. In this cohort, HbF percentage showed an HR above 1.0 but did not reach statistical significance, likely because of limited HbF availability, limited events, and lack of uniform age-adjusted HbF classification. This result should not be interpreted as contradicting prior studies. By contrast, diagnostic total hemoglobin concentration is a separate routine blood-count variable. Although it reached nominal significance in exploratory univariable Cox analysis, it is not equivalent to HbF and should not be considered a new prognostic marker.

HSCT was documented in 11 patients, and exact HSCT dates were retrieved for all 11 transplanted patients. The revised survival figure retains the Kaplan–Meier survival curve to show the observed treatment-category separation, but it explicitly labels this curve as descriptive only and separates it from the formal HSCT-related inference. To address immortal-time bias, HSCT was modeled as a post-diagnostic time-dependent covariate. In this focused sensitivity analysis, HSCT was significantly associated with better OS (HR, 0.11; 95% CI, 0.02–0.56; *p* = 0.007). This finding is consistent with the central role of allogeneic HSCT in JMML treatment, but the analysis remains observational: HSCT was not randomly assigned, occurred after diagnosis, and may reflect clinical selection, donor availability, treatment response, and survivorship before transplantation. Accordingly, the revised manuscript reports this as a statistically significant treatment-context association rather than a causal estimate of HSCT efficacy. This interpretation is aligned with prior JMML transplant studies, which establish allogeneic HSCT as the principal curative strategy while also showing substantial heterogeneity in post-transplant outcomes [31,32]. Outcomes have been evaluated in specific transplant settings, including cord blood and unrelated-donor transplantation [33,34]. Earlier multicenter experience and prospective trial data further indicate that cohort composition, diagnostic criteria, disease burden, and conditioning strategy should be considered when interpreting transplant-associated survival [35,36].

Azacitidine-based bridging or pretransplant approaches have been reported in JMML, but treatment exposure, response, and treatment timing were not uniformly documented in the present cohort and were therefore not analyzed as efficacy endpoints [37,38,39,40,41]. EFS, relapse/progression, treatment response, and cause-specific mortality were not analyzed because these outcomes were not consistently documented or uniformly adjudicated according to JMML response and outcome criteria [42,43]. Post-transplant relapse-directed approaches, including donor leukocyte infusion and second allogeneic transplantation, require relapse-defined analyses and were not evaluable in the present dataset [44,45,46].

This study has limitations. It was retrospective and single-center, with a small cohort size, limited events, and incomplete availability of methylation testing, germline-control confirmation, treatment response, cause-of-death categories, and uniformly detailed longitudinal treatment data. Molecular testing also changed over time: cases before 2017 were mainly assessed by hotspot-focused sequencing, whereas cases from 2017 onward underwent broader full-gene/next-generation sequencing. This testing-era difference may have underestimated cooperating mutations in earlier cases and is particularly relevant when interpreting PTPN11 and other non-RAS co-mutations. Germline RASopathy/Noonan-spectrum predisposition could not be uniformly excluded because matched non-hematopoietic tissue was not available for all patients. Finally, although HSCT was associated with significantly better observed OS than documented non-HSCT management, this retrospective treatment-category comparison remains susceptible to treatment-selection effects and should not be interpreted as randomized causal evidence.

In summary, KRAS-only JMML showed nominally higher monocyte and lymphocyte percentages without a higher absolute monocyte count. The NRAS/KRAS subtype alone did not significantly stratify OS. Documented PTPN11 co-mutation, including in both NRAS/KRAS co-mutated cases, highlights the importance of broader molecular context, although testing-era differences and lack of historical samples limit conclusions about clonal order. HSCT was significantly associated with better OS in the focused time-dependent Cox sensitivity analysis, but this finding should be interpreted in the context of retrospective treatment selection and post-diagnostic transplant timing rather than as randomized causal evidence.

## 5. Conclusions

In this single-center analytic cohort of children with NRAS- and/or KRAS-mutated JMML, KRAS-only disease showed nominally higher monocyte and lymphocyte percentages at diagnosis, whereas the absolute monocyte count was comparable with NRAS-only disease. The NRAS/KRAS subgroup alone did not capture the full heterogeneity of JMML. Interpretation should integrate co-mutations, testing-era limitations, germline predisposition considerations, methylation status when available, and treatment context.

Documented PTPN11 co-mutation was observed in a subset of patients and in both NRAS/KRAS co-mutated cases, but earlier historical samples were unavailable and mutation acquisition order could not be inferred. HSCT was statistically significantly associated with better OS in the focused time-dependent Cox sensitivity analysis; however, this finding should be understood as an observational treatment-context association rather than randomized evidence of transplant efficacy.

These findings support integrated JMML assessment combining RAS subgroup, age-adjusted HbF when available, DNA methylation class, co-mutations, germline predisposition evaluation, treatment response, HSCT timing, and cause-of-death annotation. Larger multicenter cohorts with uniform sequencing, germline-control testing, methylation profiling, and prospectively captured treatment timelines are required to validate and extend these observations.

## Figures and Tables

**Table 1:** Baseline clinical and hematologic characteristics by RAS subgroup.

Variable	Overall (*n* = 34)	NRAS-Only (*n* = 20)	KRAS-Only (*n* = 12)	NRAS/KRAS Co-Mutated (*n* = 2)	*p* Value *
Age at diagnosis, months	14.5 (12.0–34.8)	13.0 (12.0–26.0)	22.0 (9.0–34.5)	41.0 (36.0–46.0)	0.876
Symptom-to-diagnosis interval, months	6.0 (2.0–11.5)	6.0 (2.0–10.5)	6.5 (2.0–12.0)	2.5 (1.8–3.2)	0.984
Diagnostic total hemoglobin concentration, g/L	83.5 (77.0–97.0)	88.0 (77.0–101.8)	89.0 (77.8–95.2)	73.0 (71.0–75.0)	0.402
WBC, ×10^9^/L	20.9 (12.3–40.8)	23.0 (14.9–43.1)	18.2 (8.6–36.1)	24.9 (16.1–33.7)	0.353
Platelets, ×10^9^/L	35.5 (21.5–74.0)	56.5 (19.8–83.5)	29.0 (24.8–46.2)	44.5 (30.2–58.8)	0.459
Absolute monocyte count, ×10^9^/L	3.8 (2.1–6.3)	4.0 (2.7–5.9)	4.0 (2.5–6.7)	1.9 (1.9–1.9)	0.930
Monocytes, %	19.0 (13.5–27.7)	16.7 (11.6–24.6)	27.2 (21.6–36.3)	26.0 (26.0–26.0)	0.023
Lymphocytes, %	43.5 (36.1–46.8)	39.0 (35.8–44.4)	48.1 (45.5–56.2)	39.2 (39.2–39.2)	0.018
Reticulocytes, %	3.6 (1.8–4.7)	3.1 (1.5–4.1)	4.6 (2.5–5.7)	3.6 (3.4–3.8)	0.126
Spleen below costal margin, cm	5.0 (3.0–6.4)	5.0 (3.0–7.0)	5.6 (4.2–6.0)	4.8 (4.1–5.6)	0.967
Liver below costal margin, cm	3.0 (2.0–4.8)	3.0 (2.0–5.0)	3.0 (1.9–5.0)	2.7 (2.0–3.4)	0.830
Male sex	27/34 (79.4%)	17/20 (85.0%)	9/12 (75.0%)	1/2 (50.0%)	0.647
Lymphadenopathy	15/31 (48.4%)	11/18 (61.1%)	3/11 (27.3%)	1/2 (50.0%)	0.128
Skin rash present	15/28 (53.6%)	8/16 (50.0%)	7/10 (70.0%)	0/2 (0.0%)	0.428

* *p* values compare NRAS-only versus KRAS-only cases only. Continuous variables are median (IQR); categorical variables are *n*/available (%). Nominally significant exploratory differences were observed for monocyte percentage and lymphocyte percentage; *p* values were not adjusted for multiple comparisons.

**Table 2:** Molecular, cytogenetic, treatment, and follow-up summary.

Variable	Overall	NRAS-Only	KRAS-Only	NRAS/KRAS Co-Mutated
RAS subgroup, *n* (%)	34/34 (100.0%)	20/34 (58.8%)	12/34 (35.3%)	2/34 (5.9%)
PTPN11 co-mutation	7/34 (20.6%)	3/20 (15.0%)	2/12 (16.7%)	2/2 (100.0%)
Any non-RAS co-mutation	11/34 (32.4%)	6/20 (30.0%)	3/12 (25.0%)	2/2 (100.0%)
Monosomy 7 recorded	2/34 (5.9%)	2/20 (10.0%)	0/12 (0.0%)	0/2 (0.0%)
Abnormal karyotype recorded	4/34 (11.8%)	3/20 (15.0%)	0/12 (0.0%)	1/2 (50.0%)
Methylation tested	11/34 (32.4%)	7/20 (35.0%)	2/12 (16.7%)	2/2 (100.0%)
High methylation in full cohort	5/34 (14.7%)	3/20 (15.0%)	0/12 (0.0%)	2/2 (100.0%)
Documented HSCT exposure	11/34 (32.4%)	4/20 (20.0%)	6/12 (50.0%)	1/2 (50.0%)
OS interval available	25/34 (73.5%)	16/20 (80.0%)	9/12 (75.0%)	0/2 (0.0%)
Expanded OS analyzable	30/34 (88.2%)	18/20 (90.0%)	11/12 (91.7%)	1/2 (50.0%)
Death	15/34 (44.1%)	10/20 (50.0%)	5/12 (41.7%)	0/2 (0.0%)

Documented molecular and treatment annotations are summarized with the available denominator. Because molecular testing changed over time, unrecorded co-mutations should not be interpreted as confirmed negative findings, particularly for cases diagnosed before 2017. Percentages for the two NRAS/KRAS co-mutated cases are descriptive only and should not be used for inference.

**Table 3:** Exploratory univariable Cox proportional-hazards analysis for OS.

Variable	*n*	Events	HR	95% CI	*p* Value
KRAS-only vs. NRAS-only	29	15	0.55	0.18–1.62	0.276
Age at diagnosis, per 12 months	30	15	1.00	0.70–1.43	0.986
Symptom-to-diagnosis interval, per 6 months	30	15	1.26	0.86–1.83	0.231
Male sex	30	15	0.51	0.16–1.65	0.263
Diagnostic total hemoglobin concentration, g/L	30	15	1.74	1.13–2.69	0.012
WBC, per 10 × 10^9^/L	29	15	1.03	0.92–1.15	0.579
Platelet count, per 50 × 10^9^/L	30	15	0.69	0.35–1.37	0.290
Monocytes, per 10%	25	13	0.93	0.53–1.64	0.812
HbF percentage, per 10 percentage points	24	13	1.10	0.80–1.51	0.563
Splenomegaly, per 1 cm below costal margin	29	15	1.10	0.97–1.25	0.135
PTPN11 co-mutation	30	15	1.27	0.36–4.51	0.712
Any non-RAS co-mutation	30	15	0.82	0.26–2.58	0.733
High methylation vs. other tested	9	3	0.73	0.07–8.07	0.797

Models were fitted only when sufficient non-missing data and events were available. Hazard ratios are exploratory and unadjusted. Diagnostic total hemoglobin concentration and HbF percentage were analyzed as separate variables. HSCT exposure was not included in this Cox table because HSCT was handled as a post-diagnostic treatment-context variable and because this small retrospective cohort was not suitable for causal transplant-effect modeling.

## Data Availability

Patient-level data are not publicly available because the cohort consists of children with a rare disease and the dataset contains potentially identifiable clinical, molecular, treatment, and outcome information. De-identified data underlying the analyses may be made available from the corresponding authors upon reasonable request for peer review or research purposes, subject to institutional approval, applicable privacy requirements, and the consent/ethics restrictions governing the NICHE-Pediatrics protocol. The Supplementary Materials provide de-identified summary tables and explicit denominators for the analyses reported in this article.

## Supplementary Materials

The following supporting information can be downloaded at: https://www.mdpi.com/article/10.3390/cancers18142236/s1, Figure S1: extended biologic feature heatmap; Table S1: availability of curated clinical and laboratory variables, including follow-up and censoring denominators; Table S2: NRAS and KRAS protein-level annotations; Table S3: recorded non-RAS co-mutation counts; Table S4: privacy-minimized patient-level core clinicomolecular and outcome summary; Table S5: extended biologic and laboratory variables by RAS subgroup; Table S6: overall survival summary in the OS analysis set; Table S7: HSCT timing and time-dependent Cox sensitivity analysis.

## Abbreviations

The following abbreviations are used in this manuscript:

APTT	activated partial thromboplastin time
BFU-E	burst-forming unit–erythroid
CFU	colony-forming unit
CI	confidence interval
EFS	event-free survival
HbF	fetal hemoglobin
HR	hazard ratio
HSCT	hematopoietic stem cell transplantation
IQR	interquartile range
JMML	juvenile myelomonocytic leukemia
LDH	lactate dehydrogenase
OS	overall survival
RAS	rat sarcoma viral oncogene homolog pathway
WBC	white blood cell count
