# Supplementary material for: Clinical Phenotype, Molecular Architecture, and Survival Follow-Up in NRAS- and KRAS-Mutated Juvenile Myelomonocytic Leukemia
Source: Cancers (Basel). 2026 Jul 12;18(14):2236. doi: 10.3390/cancers18142236 (PMC13406550; doi:10.3390/cancers18142236)
Supplement: Supplementary file 1 [file cancers-18-02236-s001.zip › cancers-4376423-supplementary.pdf]

Supplementary Materials

Clinical Phenotype, Molecular Architecture, and Survival Follow-Up in NRAS- and KRAS-Mutated Juvenile Myelomonocytic Leukemia

Supplementary Materials List.

- Figure S1. Extended biologic feature heatmap.
- Table S1. Availability of curated clinical and laboratory variables.
- Table S2. NRAS and KRAS protein-level annotations.
- Table S3. Recorded non-RAS co-mutation counts.
- Table S4. Privacy-minimized patient-level core clinicomolecular and outcome summary.
- Table S5. Extended biologic and laboratory variables by RAS subgroup.
- Table S6. Overall survival summary in the OS analysis set.
- Table S7. HSCT timing and time-dependent Cox sensitivity analysis.

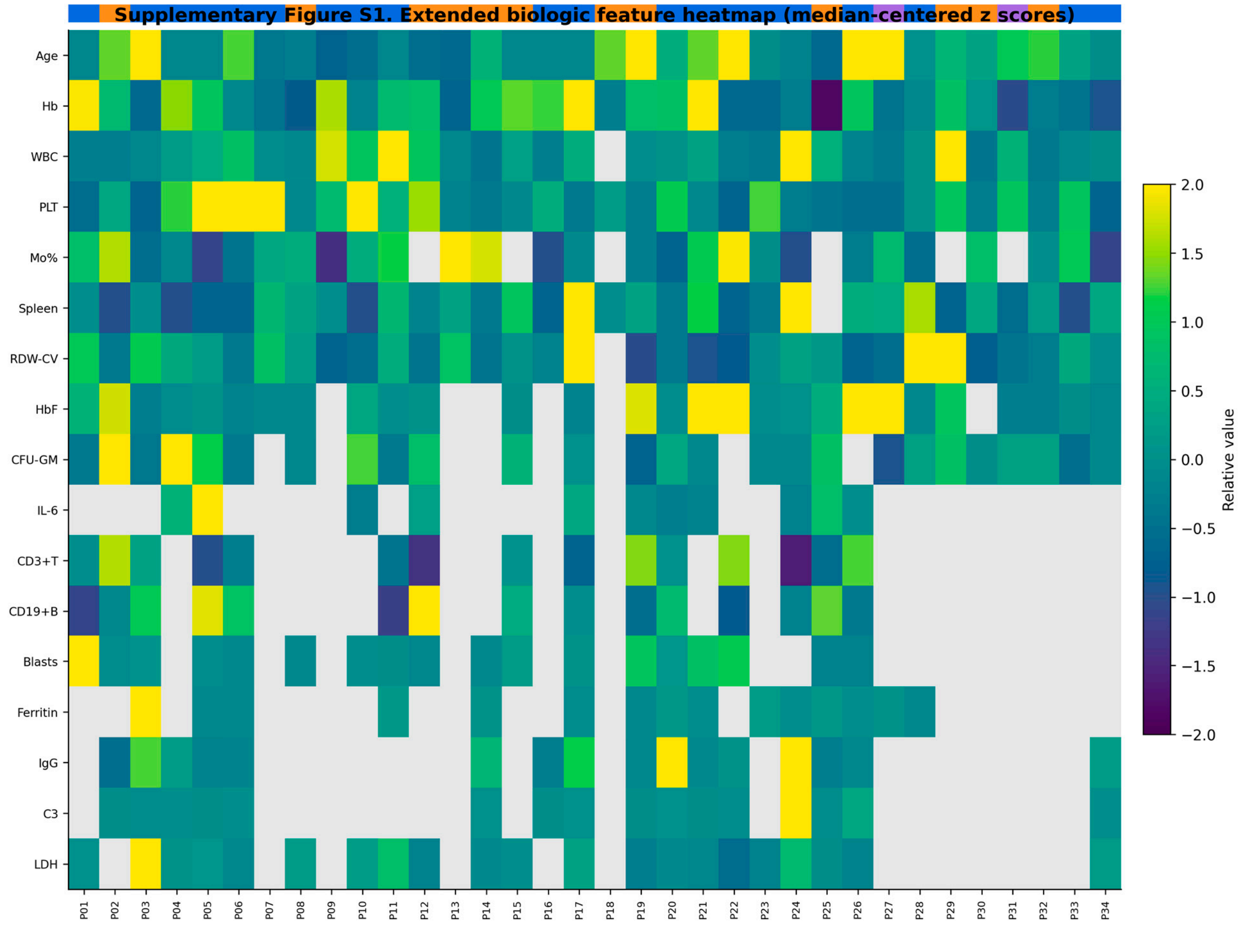

Figure S1. Extended biologic feature heatmap. Each column represents one de-identified patient, and each row represents one extended biologic or laboratory variable. Numerical variables were median-centered and scaled for visualization; the right-side color bar represents the relative z-score, with yellow indicating values above the cohort median, green/teal indicating values close to the median, and blue/purple indicating values below the median. Gray cells indicate unavailable measurements. The heatmap was used for descriptive visualization only and was not used for independent prognostic modeling.

Supplementary Tables

Table S1. Availability of curated clinical and laboratory variables. Availability is reported by variable group with patient-level values summarized as available counts and percentages. Missing values were not imputed and were not treated as normal or negative results. For molecular co-mutation annotations, recorded positive counts are distinguished from testing availability; absence of a recorded co-mutation should not be interpreted as confirmed wild-type unless explicitly stated in the source record. For HSCT, exact transplantation dates were retrieved for all documented HSCT-exposed patients and were used only for the time-dependent survival analysis.

Measurement group	Variable	Available n	Available %
Demographics/diagnosis	Sex	34	100.0%
Demographics/diagnosis	Age at diagnosis, months	34	100.0%
Demographics/diagnosis	Symptom-to-diagnosis interval, months	34	100.0%
Demographics/diagnosis	Diagnosis date	34	100.0%
Clinical phenotype	Presenting complaint	31	91.2%
Clinical phenotype	Clinical symptoms	15	44.1%
Clinical phenotype	Lymphadenopathy	31	91.2%
Clinical phenotype	Liver below costal margin	34	100.0%
Clinical phenotype	Spleen below costal margin	34	100.0%
Clinical phenotype	Rash status	28	82.4%
Core hematology	Diagnostic total hemoglobin concentration	34	100.0%
Core hematology	White blood cell count	33	97.1%
Core hematology	Platelet count	34	100.0%
Core hematology	Absolute monocyte count	32	94.1%
Core hematology	Monocyte percentage	29	85.3%
Core hematology	Absolute lymphocyte count	27	79.4%
Core hematology	Lymphocyte percentage	27	79.4%
Erythroid/colony-forming data	Reticulocyte percentage	31	91.2%
Erythroid/colony-forming data	Absolute reticulocyte count	31	91.2%
Erythroid/colony-forming data	Absolute nucleated RBC count	29	85.3%
Erythroid/colony-forming data	Nucleated RBC percentage	29	85.3%
Erythroid/colony-forming data	HbF percentage	28	82.4%
Erythroid/colony-forming data	HbF absolute value	28	82.4%
Erythroid/colony-forming data	CFU-E	26	76.5%
Erythroid/colony-forming data	BFU-E	26	76.5%
Erythroid/colony-forming data	CFU-GM	26	76.5%
Erythroid/colony-forming data	CFU-GM without GM-CSF	26	76.5%
Molecular and methylation	Gene-level mutation annotation	34	100.0%
Molecular and methylation	Protein-change annotation	28	82.4%
Molecular and methylation	PTPN11 annotation	33	97.1%

Molecular and methylation	Any recorded non-RAS co-mutation (positive annotation)	11	32.4%
Molecular and methylation	Diagnostic methylation subgroup	11	32.4%
Cytogenetics	Karyotype description	28	82.4%
Cytogenetics	Cytogenetic abnormality	22	64.7%
Cytogenetics	BCR::ABL1 status	30	88.2%
Treatment/follow-up	Treatment category documentation	31	91.2%
Treatment/follow-up	Exact HSCT date among documented HSCT cases	11/11	100.0%
Treatment/follow-up	Outcome status	28	82.4%
Treatment/follow-up	Documented OS interval	25	73.5%
Treatment/follow-up	Documented event-related interval (not a primary endpoint)	25	73.5%
Treatment/follow-up	Death date	15	44.1%
Treatment/follow-up	January 2023 follow-up documentation	5	14.7%

Table S2. NRAS and KRAS protein-level annotations. Protein-level annotations were interpreted conservatively and are reported as abstracted from the clinical molecular records. Primary subgroup analyses were based on NRAS/KRAS gene-level status rather than amino-acid hotspot categories. Noncanonical or incompletely specified changes were not used as independent prognostic variables.

Patient ID	RAS group	NRAS protein change(s)	KRAS protein change(s)	Protein-change annotation note
P01	NRAS-only	G13A	Unavailable	Interpretable RAS amino-acid change available
P02	KRAS-only	Unavailable	G12A	Interpretable RAS amino-acid change available
P03	NRAS-only	G12A	Unavailable	Interpretable RAS amino-acid change available
P04	NRAS-only	G13A	Unavailable	Interpretable RAS amino-acid change available
P05	NRAS-only	G12S	Unavailable	Interpretable RAS amino-acid change available
P06	NRAS-only	G12S	Unavailable	Interpretable RAS amino-acid change available
P07	NRAS-only	G12D	Unavailable	Interpretable RAS amino-acid change available
P08	KRAS-only	Unavailable	G13D	Interpretable RAS amino-acid change available
P09	NRAS-only	G12S	Unavailable	Interpretable RAS amino-acid change available
P10	NRAS-only	G12D	Unavailable	Interpretable RAS amino-acid change available
P11	NRAS-only	G12D	Unavailable	Interpretable RAS amino-acid change available
P12	KRAS-only	Unavailable	G12V	Interpretable RAS amino-acid change available
P13	KRAS-only	Unavailable	G13D	Interpretable RAS amino-acid change available
P14	KRAS-only	Unavailable	Unavailable	RAS gene identified; amino-acid change not unambiguously available
P15	KRAS-only	Unavailable	Unavailable	RAS gene identified; amino-acid change not unambiguously available
P16	NRAS-only	Q61H	Unavailable	Interpretable RAS amino-acid change available
P17	NRAS-only	Unavailable	Unavailable	RAS gene identified; amino-acid change not unambiguously available

P18	KRAS-only	Unavailable	Unavailable	RAS gene identified; amino-acid change not unambiguously available
P19	KRAS-only	Unavailable	G60V	Interpretable RAS amino-acid change available
P20	NRAS-only	Unavailable	Unavailable	RAS gene identified; amino-acid change not unambiguously available
P21	NRAS-only	G13D	Unavailable	Interpretable RAS amino-acid change available
P22	NRAS-only	Unavailable	Unavailable	RAS gene identified; amino-acid change not unambiguously available
P23	NRAS-only	Unavailable	Unavailable	RAS gene identified; amino-acid change not unambiguously available
P24	NRAS-only	Unavailable	Unavailable	RAS gene identified; amino-acid change not unambiguously available
P25	KRAS-only	Unavailable	G21D	Interpretable RAS amino-acid change available
P26	NRAS-only	Q61K	Unavailable	Interpretable RAS amino-acid change available
P27	NRAS/KRAS co-mutated	G12D	G13D	Interpretable RAS amino-acid change available
P28	NRAS-only	G13D	Unavailable	Interpretable RAS amino-acid change available
P29	KRAS-only	Unavailable	A59T; T58I	Interpretable RAS amino-acid change available
P30	KRAS-only	Unavailable	G13D	Interpretable RAS amino-acid change available
P31	NRAS/KRAS co-mutated	G13D; Q61H	G13D; G13-V14insG	Interpretable RAS amino-acid change available
P32	KRAS-only	Unavailable	G13D	Interpretable RAS amino-acid change available
P33	NRAS-only	Q61R	Unavailable	Interpretable RAS amino-acid change available
P34	NRAS-only	G13D	Unavailable	Interpretable RAS amino-acid change available

Table S3. Non-RAS co-mutation counts. Genes are shown as recorded positive non-RAS co-mutation annotations in the 34-patient cohort. Percentages, therefore, describe curated positive records rather than uniform whole-cohort mutation prevalence; negative or untested status should not be inferred from a blank or absent entry.

Gene	Patients	Percentage of cohort
Any recorded non-RAS co-mutation	11	32.4%
PTPN11	7	20.6%
EP300	2	5.9%
FAT1	2	5.9%
NF1	2	5.9%
ARID1A	1	2.9%
ASXL1	1	2.9%
ATG2B	1	2.9%
CBL	1	2.9%
ETV6	1	2.9%
FAM46C	1	2.9%

Gene	Patients	Percentage of cohort
FANCI	1	2.9%
FANCL	1	2.9%
GATA2	1	2.9%
HFE2	1	2.9%
IKZF1	1	2.9%
IL7R	1	2.9%
JAK3	1	2.9%
KMT2A	1	2.9%
MPL	1	2.9%
NOTCH1	1	2.9%
NOTCH2	1	2.9%
RUNX1	1	2.9%
TET2	1	2.9%
TF	1	2.9%
ZFPM1	1	2.9%

Table S4. De-identified patient-level core clinicomolecular and outcome summary. Direct patient identifiers, medical-record numbers, names, telephone numbers, full dates of birth, full follow-up dates, and free-text follow-up notes were deliberately excluded. To reduce re-identification risk in this rare pediatric disease cohort, exact patient-level ages and exact patient-level survival times are displayed as categories in this supplementary table. HSCT exposure status is shown for documentation transparency; exact transplantation dates were available for all documented HSCT-exposed patients and were used for the time-dependent Cox analysis, but exact calendar dates are not displayed in this privacy-minimized table. The exact analytic dataset is subject to institutional approval and applicable privacy restrictions.

Patient ID	RAS group	Sex	Age category, months	RAS protein change(s)	Non-RAS co-genes	Outcome/observed-time category/HSCT
P01	NRAS-only	Male	12-23	NRAS G13A	None recorded	Death; observed-time category 0-<6 mo; no HSCT documented
P02	KRAS-only	Male	24-59	KRAS G12A	None recorded	Alive/censored; observed-time category >=60 mo; no HSCT documented
P03	NRAS-only	Female	24-59	NRAS G12A	None recorded	Death; observed-time category 6-<12 mo; no HSCT documented
P04	NRAS-only	Male	12-23	NRAS G13A	None recorded	Death; observed-time category 12-<24 mo; no HSCT documented
P05	NRAS-only	Male	12-23	NRAS G12S	None recorded	Alive/censored; observed-time category >=60 mo; no HSCT documented
P06	NRAS-only	Male	24-59	NRAS G12S	None recorded	Death; observed-time category 0-<6 mo; no HSCT documented

P07	NRAS-only	Male	<12	NRAS G12D	None recorded	Alive/censored; observed-time category >=60 mo; no HSCT documented
P08	KRAS-only	Male	<12	KRAS G13D	None recorded	Death; observed-time category 12-<24 mo; no HSCT documented
P09	NRAS-only	Male	<12	NRAS G12S	None recorded	Alive/censored; observed-time category 0-<6 mo; no HSCT documented
P10	NRAS-only	Male	<12	NRAS G12D	None recorded	Alive/censored; observed-time category 12-<24 mo; no HSCT documented
P11	NRAS-only	Male	12-23	NRAS G12D	None recorded	Death; observed-time category 0-<6 mo; no HSCT documented
P12	KRAS-only	Male	<12	KRAS G12V	None recorded	Death; observed-time category 12-<24 mo; no HSCT documented
P13	KRAS-only	Female	<12	KRAS G13D	None recorded	Death; observed-time category 0-<6 mo; no HSCT documented
P14	KRAS-only	Female	24-59	RAS protein change not unambiguously available	None recorded	Alive/censored; observed-time category 6-<12 mo; HSCT documented
P15	KRAS-only	Female	12-23	RAS protein change not unambiguously available	None recorded	Death; observed-time category 12-<24 mo; no HSCT documented
P16	NRAS-only	Male	12-23	NRAS Q61H	None recorded	Death; observed-time category 6-<12 mo; HSCT documented
P17	NRAS-only	Male	12-23	RAS protein change not unambiguously available	None recorded	Death; observed-time category 6-<12 mo; no HSCT documented
P18	KRAS-only	Male	24-59	RAS protein change not unambiguously available	None recorded	Alive/censored; observed-time category 6-<12 mo; no HSCT documented
P19	KRAS-only	Male	>=60	KRAS G60V	PTPN11	Death; observed-time category 12-<24 mo; HSCT documented
P20	NRAS-only	Female	12-23	RAS protein change not unambiguously available	None recorded	Alive/censored; observed-time category 6-<12 mo; no HSCT documented
P21	NRAS-only	Female	24-59	NRAS G13D	PTPN11, FAM46C, EP300, TET2, JAK3	Death; observed-time category 0-<6 mo; no HSCT documented

P22	NRAS-only	Male	>=60	RAS protein change not unambiguously available	PTPN11, CBL	Alive/censored; observed-time category 24-<60 mo; HSCT documented
P23	NRAS-only	Male	12-23	RAS protein change not unambiguously available	None recorded	Alive/status recorded; OS interval unavailable; HSCT documented
P24	NRAS-only	Male	<12	RAS protein change not unambiguously available	GATA2, ZFPM1, FANCI, FANCL, HFE2, TF	Death; observed-time category 24-<60 mo; no HSCT documented
P25	KRAS-only	Male	<12	KRAS G21D	NOTCH2, MPL	Alive/censored; observed-time category 24-<60 mo; HSCT documented
P26	NRAS-only	Male	24-59	NRAS Q61K	PTPN11, NOTCH1, EP300, FAT1, IKZF1, IL7R	Death; observed-time category 0-<6 mo; no HSCT documented
P27	NRAS/KRAS co-mutated	Male	24-59	NRAS G12D; KRAS G13D	PTPN11, NF1	Alive/status recorded; OS interval unavailable; HSCT documented
P28	NRAS-only	Male	12-23	NRAS G13D	ASXL1, ATG2B, FAT1	Alive/status recorded; OS interval unavailable; HSCT documented
P29	KRAS-only	Male	24-59	KRAS A59T; T58I	PTPN11, NF1	Outcome unknown; OS unavailable; HSCT documented
P30	KRAS-only	Male	12-23	KRAS G13D	None recorded	Alive/censored; observed-time category 24-<60 mo; HSCT documented
P31	NRAS/KRAS co-mutated	Female	24-59	NRAS G13D; Q61H; KRAS G13D; G13-V14insG	PTPN11, ARID1A	Alive/censored; observed-time category 12-<24 mo; HSCT not recorded
P32	KRAS-only	Male	24-59	KRAS G13D	None recorded	Alive/censored; observed-time category 12-<24 mo; HSCT documented
P33	NRAS-only	Male	12-23	NRAS Q61R	KMT2A, RUNX1, ETV6	Alive/censored; observed-time category 0-<6 mo; HSCT not recorded
P34	NRAS-only	Male	12-23	NRAS G13D	None recorded	Alive/censored; observed-time category 0-<6 mo; HSCT not recorded

Table S5. Extended biologic and laboratory variables by RAS subgroup. Values are median (IQR) among patients with available measurements. Unavailable measurements were not included in the denominator and were not treated as normal results. Group-specific denominators varied across variables; these variables were summarized descriptively. No between-group p values are presented, and these subset-based variables were not used to define independent prognostic markers.

Variable	Overall	NRAS-only	KRAS-only	NRAS/KRAS co-mutated
RDW-CV, %	19.0 (17.5-20.4)	19.4 (17.7-20.9)	18.0 (17.6-19.7)	17.5 (17.4-17.5); n=2
PT, s	13.2 (12.4-15.2)	12.7 (12.4-14.7)	14.2 (13.3-15.4)	NA
APTT, s	46.7 (33.5-52.8)	37.9 (32.4-53.9)	47.1 (46.7-49.1)	NA
D-dimer	0.9 (0.6-2.3)	0.9 (0.6-3.3)	0.7 (0.7-1.5)	NA
Fibrinogen, g/L	1.84 (1.50-2.55)	1.69 (1.51-2.26)	2.63 (2.00-2.80)	NA
HbF, %	6.3 (4.5-18.8)	6.0 (4.6-11.5)	11.4 (5.6-27.4)	23.0 (12.3-33.6)
CFU-E	58.0 (29.5-69.8)	45.0 (23.0-68.0)	69.0 (52.0-80.0)	35.0 (22.5-47.5)
BFU-E	25.0 (11.5-27.0)	25.0 (16.0-26.5)	27.0 (16.0-27.0)	16.0 (9.0-23.0)
CFU-GM	15.5 (10.8-28.0)	14.0 (10.0-21.0)	25.0 (15.0-30.0)	10.5 (5.8-15.2)
CFU-GM(-)	1.0 (0.2-15.5)	5.0 (1.0-24.5)	1.0 (0.0-2.0)	0.5 (0.2-0.8)
IL-6	18.4 (8.8-41.3)	14.2 (7.1-39.4)	30.6 (21.5-43.2)	NA
TNF	24.1 (1.8-72.2)	1.9 (1.7-24.1)	150.0 (150.0-150.0)	NA
CD3+ T cells, % of lymphocytes	38.9 (27.8-53.1)	36.6 (27.8-43.2)	40.3 (28.9-64.8)	NA
CD19+ B cells, % of lymphocytes	30.8 (23.6-47.7)	28.9 (18.2-45.4)	38.8 (28.8-53.9)	NA
Myeloid blasts, %	0.9 (0.6-1.7)	1.2 (0.9-3.0)	0.6 (0.4-1.3)	NA
Mature monocytes, %	6.5 (5.1-13.9)	6.6 (5.7-16.8)	6.5 (4.2-11.0)	NA
Ferritin	49.5 (24.1-102.3)	37.0 (23.4-108.1)	68.9 (42.5-83.6)	72.4 (72.4-72.4)
EPO	20.0 (4.4-59.2)	17.3 (3.0-59.2)	22.6 (17.3-27.0)	234.6 (234.6-234.6)
IgG	13.75 (11.52-23.70)	15.65 (12.00-28.73)	10.95 (9.30-14.47)	NA
C3	0.80 (0.53-1.06)	0.81 (0.70-1.07)	0.62 (0.52-0.79)	NA
LDH	338 (265-434)	364 (266-470)	321 (257-338)	NA

Table S6. Overall survival summary in the OS analysis set. The OS analysis set comprised 25 patients with documented OS intervals and five patients with explicit January 2023 alive follow-up, censored at that date. The time column reports observed time from diagnosis to death or censoring, not a reverse Kaplan–Meier follow-up estimate. The overall observed diagnosis-to-death-or-censoring interval was 11.5 months (IQR, 4.7-19.0; range, 0.0-102.0). OS percentages should be interpreted only within the 30-patient OS analysis set and not as whole-cohort estimates.

Group	Analyzable n	Deaths	Observed time from diagnosis to death/censoring, months, median (IQR)	Median OS, months	12-month OS	24-month OS
Overall	30	15	11.5 (4.7-19.0)	13.0	53.9%	43.2%
NRAS-only	18	10	7.8 (3.2-15.0)	11.0	40.1%	40.1%
KRAS-only	11	5	13.0 (11.5-24.4)	20.0	68.2%	42.6%
NRAS/KRAS co-mutated	1	0	16.2 (16.2-16.2)	Not reached	100.0%	100.0%

Table S7. HSCT timing and time-dependent Cox sensitivity analysis. HSCT occurred after diagnosis and was therefore not treated as a fixed baseline covariate for formal inference. Exact transplantation dates were retrieved for all 11 patients with documented HSCT. The descriptive Kaplan–Meier treatment-category curve is retained only to visualize observed survival separation. The formal HSCT-related result comes from a time-dependent Cox model in which patients contributed non-HSCT risk time before transplantation and HSCT-exposed risk time after transplantation.

Analysis component	Population / denominator	Result	Formal statistic	Interpretation
Treatment documentation in full cohort	34 patients	Documented HSCT: 11; wait-and-see: 3; documented non-HSCT management: 17; treatment not documented: 3.	Not applicable	Wait-and-see and undocumented treatment categories were kept separate and were not merged into the documented non-HSCT group.
Exact HSCT timing	11 patients with documented HSCT	Exact transplantation dates retrieved for 11/11 patients. Median diagnosis-to-HSCT interval: 183 days, corresponding to 6.0 months (IQR, 140-183.5 days; 4.6-6.0 months).	Not applicable	Timing information was used to construct the post-diagnostic time-dependent HSCT exposure variable.
Descriptive treatment-category survival visualization	Patients with documented treatment category and analyzable OS	HSCT group: 2/10 deaths, median OS not reached. Documented non-HSCT management group: 13/17 deaths, median OS 10.7 months.	Kaplan–Meier curve descriptive only	The Kaplan–Meier curve visualizes observed separation but is not the formal HSCT efficacy analysis.
Formal HSCT-related survival analysis	Documented HSCT versus documented non-HSCT treatment-comparison set	HSCT was modeled as a post-diagnostic time-dependent covariate; patients contributed	HR, 0.11; 95% CI, 0.02-0.56; p = 0.007	HSCT showed a statistically significant association with better

Analysis component	Population / denominator	Result	Formal statistic	Interpretation
		non-HSCT risk time before transplantation and HSCT-exposed risk time after transplantation.		OS under time-dependent modeling.
Causal interpretation	Retrospective single-center cohort	HSCT was not randomly assigned and may reflect baseline risk, disease course, donor availability, clinical stability, treatment response, and survivorship before transplantation.	Not applicable	Report as an observational treatment-context association, not randomized causal evidence of transplant efficacy.
Reviewer-risk control	Survival analysis and supplementary reporting	HSCT is not entered as a baseline covariate in the exploratory univariable Cox table; the focused time-dependent Cox result is reported separately.	Not applicable	This handling addresses immortal-time bias while preserving transparent visualization of observed survival patterns.
